# Lazy neutrophils – a lack of DGAT1 reduces the chemotactic activity of mouse neutrophils

**DOI:** 10.1007/s00011-024-01920-6

**Published:** 2024-07-24

**Authors:** Alicja Uchańska, Agnieszka Morytko, Kamila Kwiecień, Ewa Oleszycka, Beata Grygier, Joanna Cichy, Patrycja Kwiecińska

**Affiliations:** 1 Department of Immunology, Faculty of Biochemistry, Biophysics and Biotechnology, Jagiellonian University, Cracow, Poland; 2 Present Address: Selvita S.A, Cracow, Poland; 3 Present Address: Department of Experimental Neuroendocrinology, Maj Institute of Pharmacology, Polish Academy of Science, Cracow, Poland; 4 Laboratory of Stem Cell Biology, Faculty of Biochemistry, Biophysics and Biotechnology, Jagiellonian University, Cracow, Poland

**Keywords:** DGAT1, Neutrophils, Psoriasis, Chemotaxis, Lipids, Retinoic acid

## Abstract

**Background:**

Neutrophils are key players in the innate immune system, actively migrating to sites of inflammation in the highly energetic process of chemotaxis. In this study, we focus on the role of acyl-CoA: diacylglycerol acyltransferase 1 (DGAT1), an enzyme that catalyzes the synthesis of triglycerides, the major form of stored energy, in neutrophil chemotaxis.

**Methods and results:**

Using a mouse model of psoriasis, we show that DGAT1-deficiency reduces energy-demanding neutrophil infiltration to the site of inflammation, but this inhibition is not caused by decreased glycolysis and reduced ATP production by neutrophils lacking DGAT1. Flow cytometry and immunohistochemistry analysis demonstrate that DGAT1 also does not influence lipid accumulation in lipid droplets during inflammation. Interestingly, as has been shown previously, a lack of DGAT1 leads to an increase in the concentration of retinoic acid, and here, using real-time PCR and publicly-available next-generation RNA sequencing datasets, we show the upregulation of retinoic acid-responsive genes in *Dgat1*KO neutrophils. Furthermore, supplementation of WT neutrophils with exogenous retinoic acid mimics DGAT1-deficiency in the inhibition of neutrophil chemotaxis in in vitro transwell assay.

**Conclusions:**

These results suggest that impaired skin infiltration by neutrophils in *Dgat1*KO mice is a result of the inhibitory action of an increased concentration of retinoic acid, rather than impaired lipid metabolism in DGAT1-deficient mice.

**Supplementary Information:**

The online version contains supplementary material available at 10.1007/s00011-024-01920-6.

## Introduction

Human neutrophils account for the majority of peripheral blood leukocytes, yet knowledge of their biology remains obscure. For decades, neutrophils were described as short-lived, transcriptionally nonactive and terminally differentiated cells. In recent years it has been shown that mature neutrophils demonstrate tissue-dependent transcriptional, functional and metabolic plasticity [1, 2]. Neutrophils serve an essential function in the innate immune system, as they are the first cells to be recruited into infected tissues in order to provide immune protection [3]. On the other hand, they also contribute to the pathophysiology of some autoimmune diseases, such as psoriasis [4]. Neutrophils represent a numerically dominant and characteristic component of the inflammatory infiltrate in psoriasis, but their role in the pathogenesis of this disease is still not clear.

It is well documented that neutrophils are primarily glycolytic cells [5], containing fewer and less active mitochondria than other immune cells, mainly to maintain their mitochondrial membrane potential for apoptotic signaling [6]. Resting neutrophils accumulate triacylglycerols (TGs) [7], which are neutral lipids and the major form of stored energy in mammals. The cytoplasmic storage of neutral lipids, including TGs, in lipid droplets (LDs) serves to provide reservoirs for membrane formation and maintenance, regulate cellular lipid homeostasis and signaling, detoxify fatty acids, serve as fuel in times of stress or nutrient deprivation, and contribute to immunity [8].

During inflammatory responses, LDs from neutrophils become more prominent in number and size [9]. On the other hand, the generation of free fatty acids derived from the lipolysis of LDs via autophagy is essential for neutrophil development from myeloblasts [10]. It has also been suggested that the lipid alterations in neutrophils may modify the physiology of these cells, and therefore, the host defense mechanisms [11].

TGs can be broken down into fatty acids and glycerol, then converted into dihydroxyacetone phosphate, which enters glycolysis. The last step of TGs synthesis in mammals is catalyzed by enzymes with acyl-CoA: diacylglycerol acyltransferase (DGAT) activity. There are two known proteins with DGAT activity, namely DGAT1 and DGAT2 [12, 13]. Both are ubiquitously expressed in mammalian tissues with the highest levels of expression found in tissues that are active in TGs synthesis, such as adipose tissue, the small intestine, liver and mammary gland. Notably, DGATs are also found in leukocytes, with the highest expression level noted in neutrophils (https://www.immgen.org). Both DGAT1 and DGAT2 are characterized by acyl-CoA: diacylglycerol acyltransferase activity. However, these enzymes share no sequence homology and are members of different gene families with distinct biochemical, cellular and physiological functions [14]. Mice with a genetic deficiency in *Dgat1* provide evidence that DGAT1 plays an important role in the regulation of energy metabolism [15, 16]. DGAT1, in addition to catalyzing the synthesis of TGs, also shows acyl-CoA: monoacylglycerol acyltransferase, wax synthase and acyl-CoA: retinol acyltransferase (ARAT) activities [17]. DGAT1 converts retinoic acid (RA) into retinyl esters, and thereby maintains retinoid homeostasis [18].

RA is a potent signaling molecule, involved in the regulation of a wide range of physiological processes, including immune responses. It plays a crucial role during the process of neutrophil maturation and differentiation [19]. Disorders in retinoid homeostasis and signaling can lead to a block in the differentiation of myeloid lineage which is likely the result of repressing the expression of RA responsive genes [20]. Therapeutic administration of RA regulates cytokine expression and has anti-inflammatory effects in dermatological diseases, such as psoriasis, at least partially through the modulating activity of the neutrophils [21]. It has long been known that RA inhibits chemotactic responses, superoxide anion production and lysosomal enzyme release by neutrophils [22–24].

Given that DGAT1 is responsible for catalyzing the synthesis of TGs and conversion of RA, we hypothesize that this enzyme modulates metabolism and the activity of neutrophils by controlling the bioavailability of these compounds. In this study we use the mouse model of psoriasis to elucidate the influence of DGAT1 on neutrophil activity in chronic inflammation.

## Materials and methods

### Mice

All animal procedures and experiments were performed in accordance with national and European legislation, after approval by the 2nd Local Institutional Animal Care and Use Committee (IACUC) in Cracow (approval #320/2018 with subsequent supplementary approval #80/2021). The *Dgat1* knock-out mice – B6.129S4-Dgat1^tm1Far^/J (strain 003824) were obtained from The Jackson Laboratory and housed under specific pathogen-free conditions in the animal facility at the Faculty of Biochemistry, Biophysics and Biotechnology of the Jagiellonian University in Cracow. The mice used in this study were sex-matched 8- to 12-week-old littermates.

### Psoriasis-like skin dermatitis induction and tissue preparation

An imiquimod (IMQ) model of psoriasis was induced as previously described [25]. All mice were analyzed at 8 weeks of age because of the dynamic skin changes observed after this time point [18]. The mice were treated twice a day for up to 6 days with 15 mg of Aldara^™^ cream (5% imiquimod) (Meda AB) or Vaseline (Unilever) on 1 cm^2^ shaved and depilated back skin. At the end of the procedure, the mice were sacrificed by an overdose of anesthetics and the tissues were isolated.

The 0.25-cm^2^ skin biopsies were mounted and frozen in cryo-matrix in liquid nitrogen vapors, and stored in -80 °C for immunohistochemistry. Blood samples were collected by retro-orbital bleeding into tubes containing 10 mM EDTA (Sarstedt). Bone marrow cells were isolated from one femur, as described previously [26]. 0.5-cm^2^ back skin biopsies were cut into small pieces using scissors and enzymatically digested using the Multi Tissue Dissociation Kit 1 (Miltenyi Biotech), continuously shaking (1400 rpm) at 37 °C for 30 min. Single-cell suspensions from the skin, spleen, and bone marrow were obtained by mashing through 40-µm cell strainers. Red blood cells were removed using red blood cell lysis buffer (eBioscience) and the cells were resuspended in an RPMI1640 medium (Biowest) supplemented with 2% heat-inactivated FBS (Gibco), and immediately stained for flow cytometry analysis.

### Preparation of skin extracts

Psoriatic-like skin biopsies were acquired from WT and *Dgat1*KO mice on the 3rd day of IMQ treatment. The fragmented skin sections were suspended in RPMI1640 medium (Biowest) supplemented with 10% FBS (Gibco) (1 mg of tissue in 20 µl of medium) and incubated for 6 h at 37 °C. Skin extracts were obtained by mashing the samples through 40-µm cell strainers. The extracts were stored at -20 °C until use.

### Neutrophil isolation

Blood was collected by cardiac puncture to a syringe containing 3.2% (w/v) sodium citrate (Sigma-Aldrich), and diluted (1:5) in PBS (PAN Biotech) containing 0.5% BSA (Sigma-Aldrich). Specific cell populations from whole blood were isolated by density gradient centrifugation. Diluted blood was layered on top of the density gradient separation solutions (Pancoll human for Granulocytes 1.119 g/ml, Pancoll human 1.077 g/ml and blood in a volume ratio 1:1:2) (PAN Biotech). After centrifugation and wash, the remaining red blood cells in the granulocyte fraction were lysed by pyrogenic-free water. Then the cells were resuspended in RPMI1640 medium (Biowest) supplemented with 10% heat-inactivated FBS (Gibco).

Bone marrow neutrophils were isolated as described previously [26] from femurs and tibias by 10,000 × g centrifugation. After red blood cell lysis, the neutrophils were negatively isolated using a Neutrophil Isolation Kit, mouse (Miltenyi Biotec) and LS MACS columns (Miltenyi Biotec), according to the manufacturer’s instructions.

The purity of the isolated cells were examined by flow cytometry based on CD45, CD11b and Ly6G immunoreactivity. The neutrophils used for further assays were > 95% pure.

### In vitro chemotaxis assay

The neutrophil chemotaxis assay was performed using transwell 24-well plates with 3.0-µm pore polycarbonate membrane inserts (Corning Costar). Peripheral blood neutrophils were resuspended in RPMI 1640 medium supplemented with 10% heat-inactivated FBS. The cells were added into the upper wells (200,000 cells/well), and the culture medium with 100 nM leukotriene B_4_ (LTB_4_) (Sigma-Aldrich), 100 nM all-*trans* retinoic acid (atRA) (Sigma-Aldrich) or skin extract, diluted 5 times, from IMQ-treated WT and *Dgat1*KO mice were added to the lower wells. Compounds that modulate cell metabolism were added to the upper and lower wells: 50 mM 2-deoxy-D-glucose (2-DG) (Merck), 0.5 µM rotenone (Agilent) and 0.5 µM antimycin A (Agilent). In some experiments, resuspended neutrophils were pretreated with vehicle or atRA (100 nM) for 2 h. The plates were incubated at 37 °C for 1 h. After removing the upper wells, the number of migrated cells was determined by flow cytometry.

### LTB_4_ concentration measurement

The concentration of LTB_4_ in the culture medium conditioned with skin extract from IMQ-treated WT and *Dgat1*KO mice was measured using commercially available LTB_4_ ELISA kit (Enzo Life Sciences) according to the manufacturer’s instructions.

### Fatty acids uptake

Bone marrow neutrophils were cultured in HBSS (Gibco) supplemented with 200 µM oleic acid-BSA complex (Sigma-Aldrich) or 1% fatty acid-free BSA (Sigma-Aldrich) as a control, for 3 h in 37 °C. LDs were quantified by flow cytometry and visualized by histochemistry, as described below.

### Immunohistochemistry

Immediately after isolation, enriched bone marrow neutrophils were deposited on microscope slides (18 × g, 10 min) using a cytospin cytocentrifuge. The cells were then fixed with 3.7% formaldehyde, and the lipid droplets were stained using 2 µM BODIPY 493/503 (ThermoFisher Scientific), and counterstained with Hoechst 33258 (Invitrogen) to visualize DNA.

Frozen 10-µm skin sections from WT and *Dgat1*KO mice were fixed in ice-cold acetone and stained with rat anti-mouse Ly6G PE-conjugated (eBioscience) antibodies or rat anti-mouse IgG2a K PE-conjugated isotype control (eBioscience). The sections were counterstained with Hoechst dye 33258 (Invitrogen). The slides were mounted using Fluorescence Mounting Medium (Dako). Images were captured with a fully motorized fluorescence microscope (Nikon, Eclipse) and analyzed using the software NIS-Elements (Nikon).

### Flow cytometry

The cell suspensions were stained for viability assessment (Zombie Aqua Fixable Viability Kit; BioLegend). Intracellular LDs were quantified by staining with the fluorescent dye BODIPY 493/503 (ThermoFisher Scientific). Then unspecific antibody-binding sites were blocked with anti-CD16/CD32 antibodies (Fc block; eBioscience) followed by staining with directly conjugated antibodies: CD45.2-APC/Cy7 (clone HI30, BioLegend), CD11b-eFluor450 (clone M1/70, eBioscience), Ly6G-APC (clone 1A8, BioLegend), c-Kit-PE/Cy7 (clone 2B8, BioLegend), Lin-AF700 (BioLegend), CD16/32-PE (clone 2.4G2, BD Biosciences), c-Kit-APC/eF780 (clone 2B8, eBioscience), CD34-FITC (clone RAM34, eBioscience), Sca-1-PE/Cy7 (clone D7, BD Biosciences), CD19-PerCP/Cy5.5 (clone 1D3, eBioscience), CD3-AF594 (clone 17A2, BioLegend), Ly6G-BV711 (clone 1A8, BD Biosciences), BST-1-PE (clone BP-3, BioLegend). After staining, the cells were fixed with 4% paraformaldehyde. Data were acquired using a BD LSRII (BD Biosciences), and were analyzed using FCS Express (De Novo Software).

Singlets were selected on the basis of FCS-A vs. FCS-H. Dead cells were routinely excluded from the analysis. The frequencies of specific cell types were calculated as the percentage of CD45^+^ cells. Neutrophils were defined as live, CD45^+^, CD11b^+^ and Ly6G^+^ cells. Neutrophil progenitors were defined according to the gating strategy described previously [27]. The lineage antibody cocktail included anti-CD3 (clone 17A2), anti-Ly-6G/Ly-6 C (clone RB6-8C5), anti-CD11b (clone M1/70), anti-B220 (clone RA3-6B2) and anti-TER-119 (clone Ter-119) antibodies (BioLegend). C-Kit and CD34 were used to exclude CD34– c-Kit^+^ stem/progenitor cells and FSC/SSC gating excluded eosinophils. “Fluorescence minus one” (FMO) controls were routinely used to set the thresholds for positive/negative events.

### In vivo neutrophil activation (sodium periodate–induced peritoneal inflammation)

Sterile peritonitis was induced in WT and *Dgat1*KO mice by intraperitoneal injection of 1 ml of 5 mM sodium meta-periodate (Sigma-Aldrich) [28]. After 4 h, the mice were sacrificed and the peritoneal cells were harvested by lavage with PBS with 2 mM EDTA (Sigma-Aldrich). The total lavage cells were plated in tissue-culture plates in RPMI1640 medium (Biowest) supplemented with 10% heat-inactivated FBS (Gibco) for 2 h. The purity of the nonadherent cells (neutrophils) were examined by flow cytometry based on CD45, CD11b and Ly6G immunoreactivity. Samples with > 98% purity were used for the gene expression analysis.

### Real-time PCR

Skin biopsies were immediately placed to Stay-RNA (A&A Biotechnology), and total RNA were extracted using Total RNA Zol-Out kit (A&A Biotechnology) and converted to cDNA using NxGen M-MulV reverse transcriptase (Lucigen) with random primers (Invitrogen) and oligo(dT) (Genomed). Real-time PCR was performed on the CFX96 Real-Time System (Bio-Rad) using RT HS-PCR Mix SYBR containing universal PCR master mix (A&A Biotechnology) and primers specific for a mouse: *Gapdh* (5’- TGTGTCCGTCGTGGATCTGA, 5’ - TTGCTGTTGAAGTCGCAGGAG), and *Ltb4r1* (5’- GGGAAACCCTGTCCTTTTGAT, 5’- CCTTTTCAGGATGCTCCACAC). The levels of mRNA expression in each sample were analyzed in duplicate, normalized to the housekeeping gene *Gapdh* and shown as a relative expression (calculated using the 2^−ΔΔCt^ method) [29].

Additional RA-responsive genes were selected by analysis of publicly available bulk RNAseq data from the Immunological Genome Project (GSE109125) [30]. Records with sorted neutrophils (GN.BM, GN.Sp, GN.Thio.PC) were searched for high expression of genes listed as upregulated by retinoic acid, based on previous studies [31] and their gene ontology description. Genes chosen for further qPCR analysis were: *Bst1* (5’- CCTATCCCACGAGAGGGTTT, 5’- GGGCCTCCAATCTGTCTTCC), *Ptafr* (5’- AGCTCCTCCTACAGGCATATT, 5’- TCGGAAAGAGCGTGTATCGAA) and *Fgr* (5’- ACCCCCAACAAGGAACCAAG, 5’- ATCCCAAAGGACCACACGTC).

### Attachment of neutrophils to the assay plates for bioenergetic assessment

Cellular bioenergetics were determined after the peripheral blood neutrophils had been seeded to 8-well polystyrene plates designed for the extracellular flux analyzer. The purified neutrophils were resuspended in sterile XF assay buffer (Agilent Seahorse XF Base Medium Minimal DMEM supplemented with 1 mM sodium pyruvate, 2 mM L-glutamine, 10 mM D-glucose, pH 7.4), and seeded (2 × 10^5^ cells/well) in 80 µl to the CellTak (BD Biosciences)-coated assay plates. Then the cells were attached to the bottom of the plate by centrifugation at 45 × g for 1 min without the brake, brought up to 180 µl using XF assay buffer and allowed to adjust for 30 min at 37 °C without CO_2_.

### Glycolytic rate measurement

The glycolytic rate of the murine neutrophils was determined using the Seahorse XF bioanalyzer (Agilent Technologies). After the initial assessment of the basal proton efflux rates (PER), sequential exposures to modulators of the glycolytic pathway were injected at optimized concentrations using a standard glycolytic rate assay test. First, the mitochondrial electron transport chain was inhibited by adding rotenone (0.5 µM) and antimycin A (0.5 µM) to inhibit mitochondrial oxygen consumption and show compensatory glycolysis. Then, the glycolysis was inhibited by injecting 2-deoxy-D-glucose (2-DG) (50 mM), a glucose analog which inhibits glycolysis through competitive binding of glucose hexokinase, the first enzyme in the glycolytic pathway. After each experiment, PER data were normalized to the corresponding cell numbers in each well using protein determination (BCA assay).

### ATP measurement

Bone marrow neutrophils from WT and *Dgat1*KO mice were plated 1 × 10^5^ cells/well on a 96-well white plate with a transparent bottom for luminescence assays and cultured with 50 mM 2-DG for 1 h at 37 °C. Then 100 ng/ml PMA was added for 15 min to activate the neutrophils. The ATP level was determined using an ADP/ATP Ratio Assay Kit (Sigma-Aldrich), according to the manufacturer’s instruction.

### Statistical analysis

The statistics were analyzed using GraphPad Prism 9 (GraphPad Software). The data are shown as mean ± standard error of the mean (SEM). The specific tests performed and number of samples per group are described in the figure legends.

## Results

### DGAT1-deficiency reduces neutrophil infiltration into IMQ-induced psoriatic skin

**Fig. 1:**
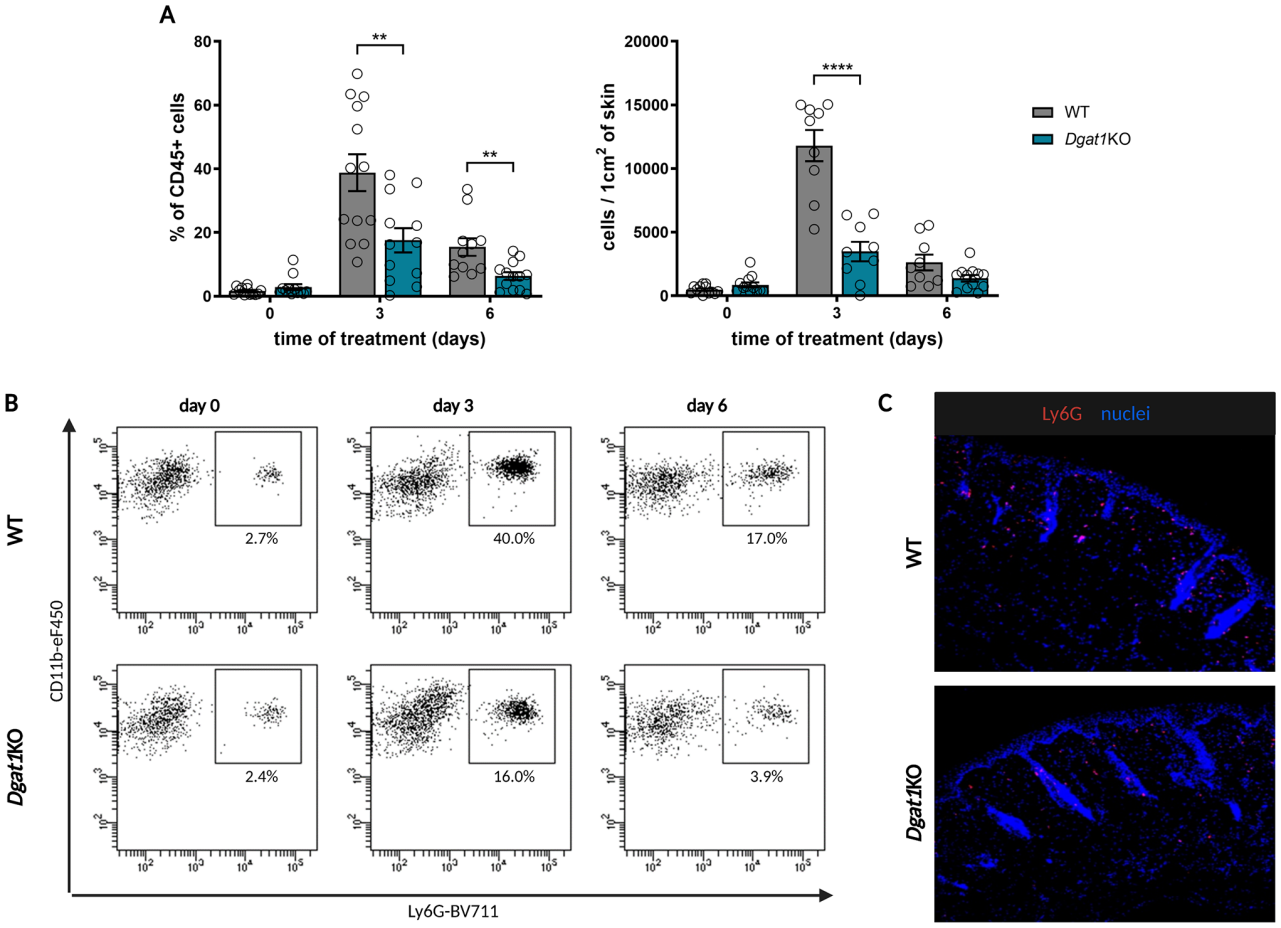
Lack of DGAT1 results in inhibited neutrophil infiltration of lesional skin in an experimental model of psoriasis. WT and *Dgat1*KO mice were treated with IMQ to induce psoriasis-like dermatitis. **(A)** Skin biopsies were harvested and subjected to flow cytometry analysis. Total leukocytes were detected using anti-CD45 antibodies, whereas neutrophils were detected using anti-Ly6G and anti-CD11b antibodies. Data are shown as a percentage of neutrophils among CD45^+^ cells (left panel) and as absolute number of neutrophils in 1 cm^2^ of skin (right panel). The data are shown as a mean of *n* = 11–13 mice ± SEM. Gray bars = WT mice; turquoise bars = *Dgat1*KO mice. ** *p* < 0.01, **** *p* < 0.0001 by two-way ANOVA, Tuckey post hoc test. **(B)** Representative flow cytometry plots of the neutrophils are shown. **(C)** Skin biopsies were harvested and analyzed using histology. Fluorescence microscopy images of murine lesional skin stained for Ly6G (red) and DNA (blue). Results are representative of at least three independent experiments

The epidermal accumulation of neutrophils and the formation of Munro’s microabscesses are histological hallmarks of psoriasis in humans [32], and are also observed in the IMQ-induced mouse model of psoriasis [33, 34]. Kinetic analysis revealed that neutrophil recruitment into IMQ-treated skin of WT mice is at its highest on the 3rd day of treatment (Suppl. Figure 1). Therefore, this model was selected to study the involvement of DGAT1 in neutrophil accumulation and function in chronic inflammatory disease. Flow cytometry and immunohistochemistry analysis of psoriasis-like skin demonstrated a markedly lower number of these cells in *Dgat1*KO mice in comparison to WT littermates (Fig. 1), suggesting an impaired DGAT1-mediated neutrophil infiltration into psoriatic skin. Importantly, the difference in neutrophil number was observed only at the site of inflammation but not systemically, as immunophenotyping of peripheral blood, spleen and bone marrow at days 0, 3 and 6 revealed comparable neutrophil counts between WT and *Dgat1*KO mice (Suppl. Figure 2 A). Moreover, the lack of DGAT1 did not influence granulopoiesis (Suppl. Figure 2 C).

**Fig. 2:**
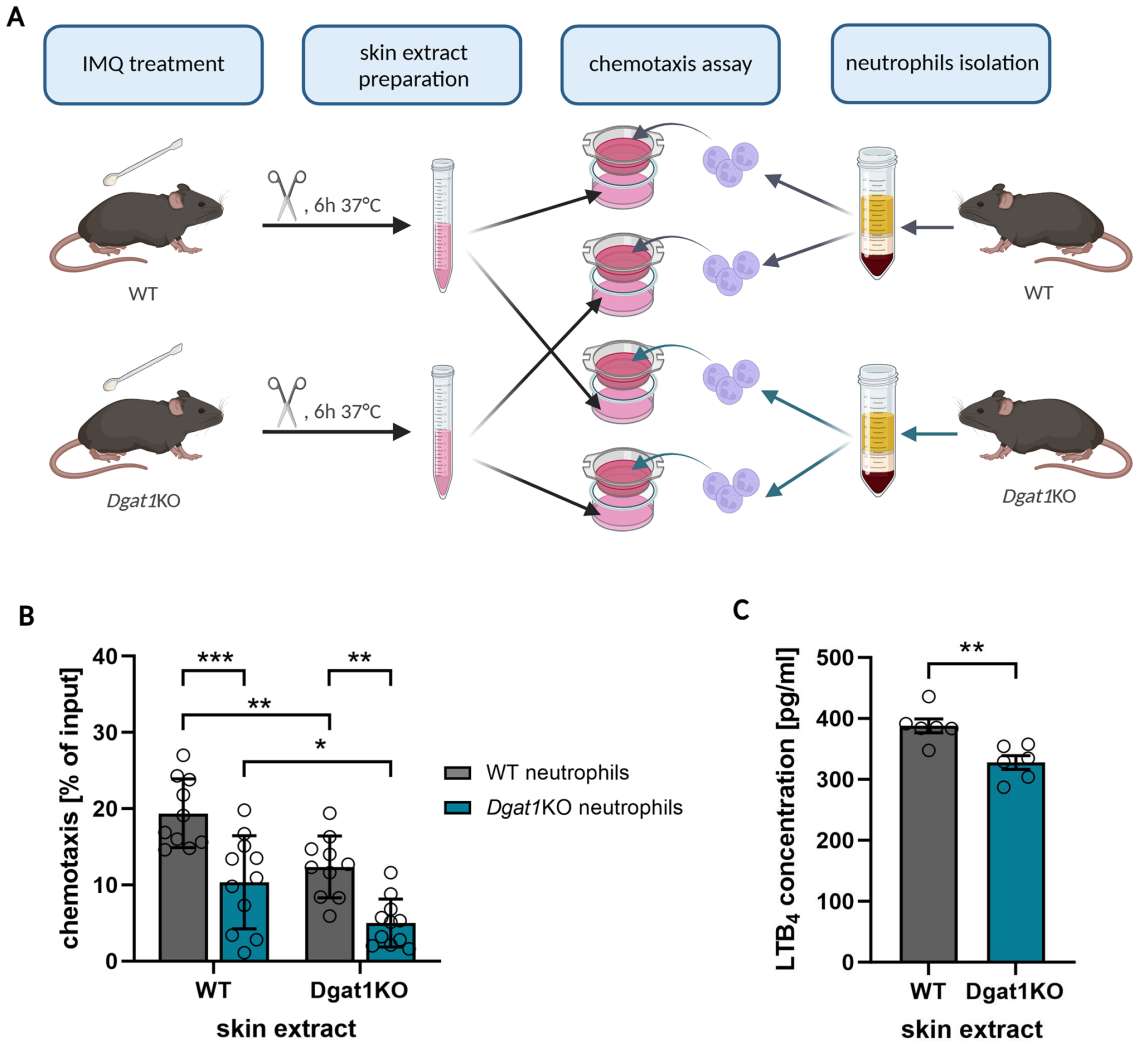
Inhibited neutrophil recruitment to the lesional skin of *Dgat1*KO mice is a mixed effect of neutrophils and the skin. **(A)** Schematic presentation of chemotaxis assay with skin extracts from IMQ-treated mice. **(B) ** In vitro transwell chemotaxis assay for peripheral blood WT and *Dgat1*KO neutrophils to skin extracts from IMQ-treated WT and *Dgat1*KO mice. The data are shown as a mean of *n* = 10 ± SEM. * *p* < 0.05, ** *p* < 0.01, *** *p* < 0.001 by two-way ANOVA, Tuckey post hoc test. (**C**) LTB_4_ concentration measured in media with skin extract from IMQ-treated WT and *Dgat1*KO mice. The data are shown as a mean of *n* = 6 ± SEM. ** *p* < 0.01 by t-test

These results raise the question as to whether lower neutrophil accumulation in IMQ-treated skin depends on neutrophils or is a consequence of the altered skin microenvironment in *Dgat1*KO mice. As was shown previously, adult mice with a DGAT1 deficiency develop fur lipid abnormalities, sebaceous gland atrophy, hair loss, and increased transepidermal water loss [18, 35, 36]. Therefore, it was possible that the altered skin environment in *Dgat1*KO mice could impact on neutrophil infiltration during inflammation. To establish how DGAT1 deficiency influences the migratory behavior of neutrophils we performed chemotaxis assay in vitro with peripheral blood neutrophils from *Dgat1*KO and WT mice, and skin extracts as a source of chemoattractants. The psoriatic skin environment of WT and *Dgat1*KO mice was mimicked by skin extracts from both WT and *Dgat1*KO mice with induced psoriasis-like dermatitis (Fig. 2A). We observed significantly reduced chemotaxis of the neutrophils from the *Dgat1*KO mice compared to WT mice when they are attracted to the skin extracts derived from either WT and *Dgat1*KO mice (Fig. 2B). We also noticed significantly less robust migration of neutrophils to the skin extracts from *Dgat1*KO mice compared to the WT mice, independently of the genotype of the neutrophils. Taken together, these data suggested that the lesser infiltration to the skin of *Dgat1*KO mice by neutrophils is a combined effect of the reduced migratory capacity of neutrophils lacking DGAT1 as well as the less-supportive cutaneous environment of *Dgat1*KO mice.

Since DGAT1 is involved in lipid metabolism, we next investigated whether lipid chemoattractants could be responsible for differential neutrophil migration in the absence of this enzyme. Leukotriene B_4_ (LTB_4_) is the strongest lipid chemoattractant for neutrophils, playing a key role in neutrophil recruitment in the early stages of inflammation and at the amplification stage [37–39]. Furthermore, a potential involvement of LTB_4_ in the skin recruitment of neutrophils in this model was supported by a previous report indicating that the LTB_4_ receptor BLT1 (also known as LTB_4_ receptor 1 [*Ltb4r1*]) is upregulated during IMQ-induced skin inflammation [39].

**Fig. 3:**
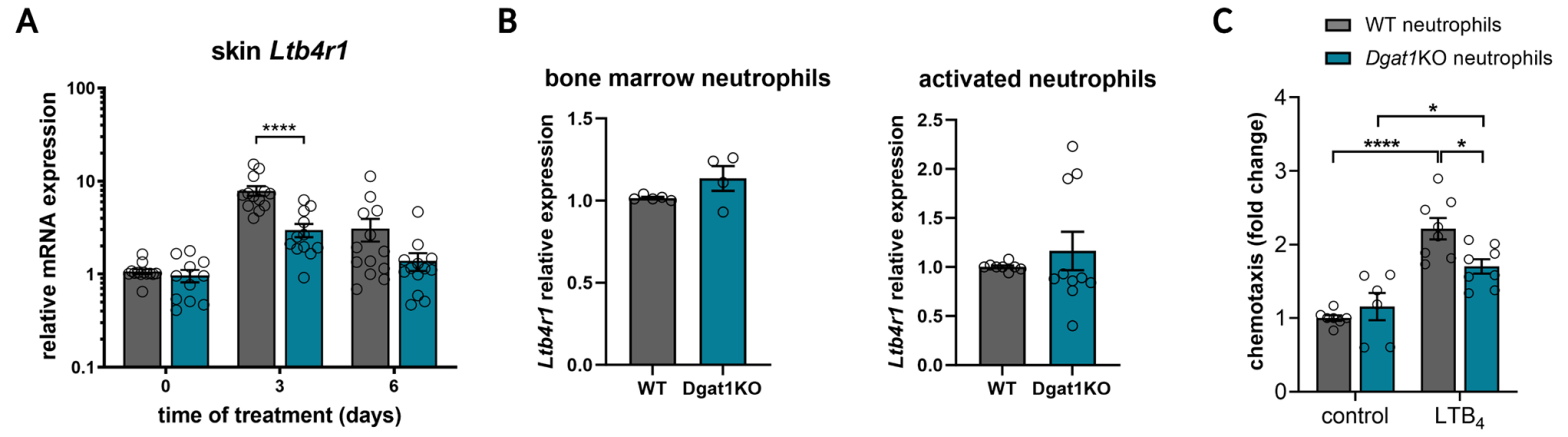
LTB_4_-inducible chemotaxis of neutrophils is inhibited by all-*trans* retinoic acid. **(A) ***Ltb4r1* gene expression changes during IMQ treatment of WT and *Dgat1*KO mice. The data are shown as a mean of *n* = 11–13 mice ± SEM. Gray bars = WT mice; turquoise bars = *Dgat1*KO mice. **** *p* < 0.0001 by two-way ANOVA, Tuckey post hoc test. **(B) ***Ltb4r1* gene expression in bone marrow neutrophils (*n* = 4–5) and activated neutrophils from sterile peritonitis (*n* = 8–10). The data are shown as a mean ± SEM; statistics were analyzed using *t*-test. **(C) ** In vitro transwell chemotaxis assay for peripheral blood WT and *Dgat1*KO neutrophils with 100 nM leukotriene B_4_ (LTB_4_) as a chemoattractant. The data are shown as a fold change related to control WT neutrophils. The mean of *n* = 6–8 ± SEM is shown. * *p* < 0.05, **** *p* < 0.0001 by two-way ANOVA, Tuckey post hoc test

In the media conditioned with skin extract from *Dgat1*KO mice, we observed a significantly lower concentration of LTB_4_ compared to the media with WT skin extract (Fig. 2C). This observation was further supported by the analysis of gene expression of key enzymes involved in LTB_4_ synthesis – *Alox5* and *Lta4h*, indicating a down-regulation of *Alox5* expression in the skin of *Dgat1*KO mice compared to WT mice (Suppl. Figure 3A). Together, these results suggest that the downregulated levels of LTB_4_ could contribute to the observed reduction in neutrophil influx into the skin of *Dgat1*KO mice. However, it was also plausible that the reduced chemotactic ability of neutrophils in *Dgat1*KO mice, possibly due to their altered levels of BLT1, plays a role in this phenomenon. In agreement with a previous report [39], we observed increased gene expression of the LTB_4_ receptor BLT1 during IMQ-induced skin inflammation. Importantly, we also found that *Ltb4r1* expression is significantly down-regulated in the skin of *Dgat1*KO mice (Fig. 3A). Since mice lacking *Ltb4r1* expression show attenuated neutrophil infiltration into IMQ-induced psoriatic skin [39], we next investigated the level of *Ltb4r1* expression in neutrophils from WT and *Dgat1*KO mice. However, there was no significant difference in *Ltb4r1* expression in naïve neutrophils isolated from bone marrow. Likewise, activated neutrophils from the peritoneum of WT and *Dgat1*KO mice did not differ in *Ltb4r1* expression levels either (Fig. 3B). These results, together with microarray and RNA-seq data from murine leukocytes showing the highest expression of *Ltb4r1* in neutrophils (https://www.immgen.org), suggest that lower *Ltb4r1* expression in the skin of *Dgat1*KO mice is directly related to lower neutrophil recruitment to the lesional skin. The impact of DGAT1 on LTB_4_ mediated neutrophil migration was investigated by the transwell chemotaxis assay in which we observed significantly inhibited LTB_4_-induced chemotaxis of *Dgat1*KO neutrophils compared to WT neutrophils (Fig. 3C). Taken together, these results suggest that lack of DGAT1 in neutrophils weakens their migratory response to chemoattractants such as LTB_4_.

### DGAT1 does not affect LD formation and glycolysis in neutrophils, but potentially increases retinoic acid concentration

**Fig. 4:**
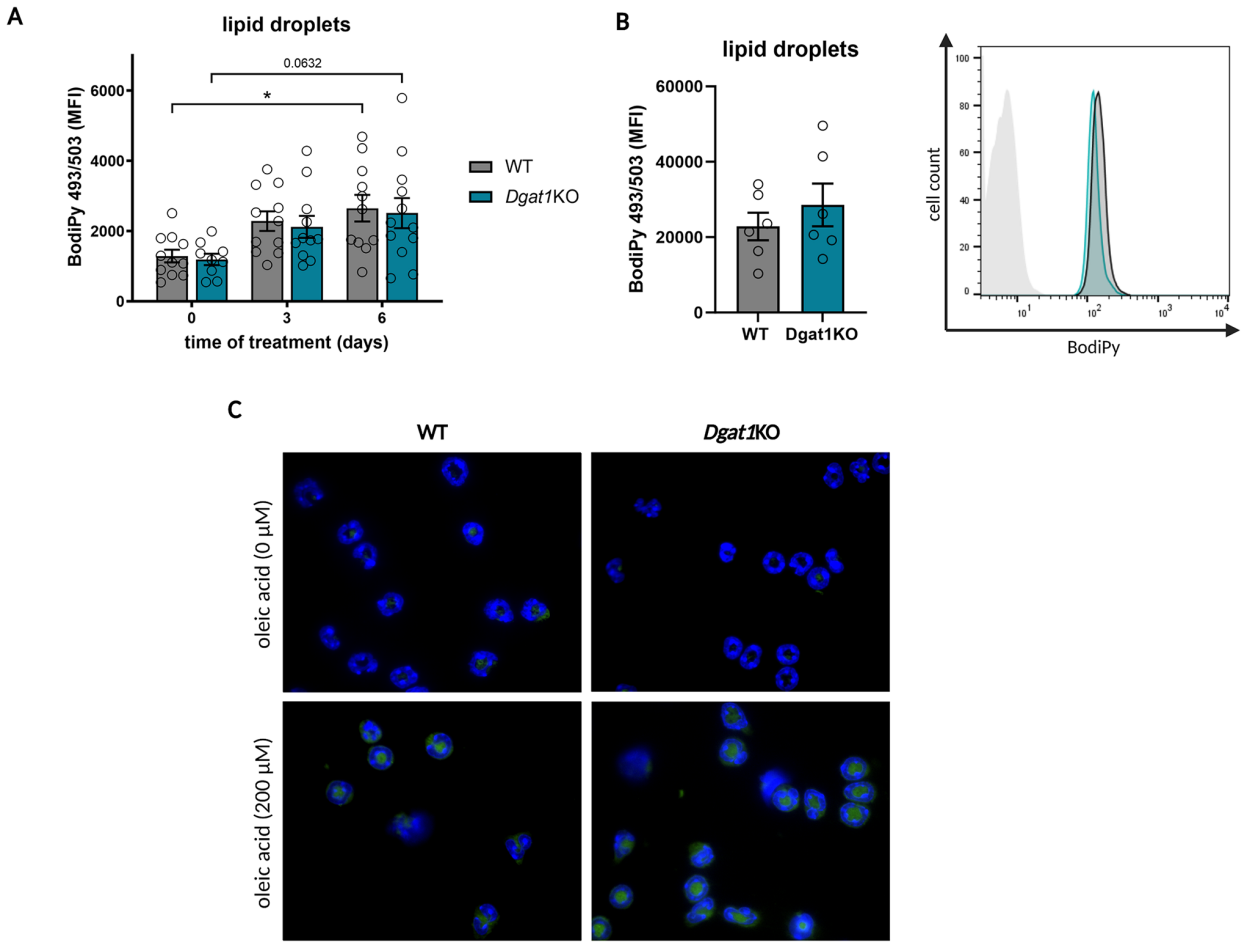
Lipid droplets accumulation in mouse neutrophils is DGAT1-independent. **(A)** Accumulation of lipid droplets in neutrophils infiltrating IMQ-treated skin of WT and *Dgat1*KO mice. Skin biopsies were harvested and subjected to flow cytometry analysis. Neutrophils were detected using anti-CD45, anti-Ly6G and anti-CD11b antibodies. Lipid droplets were stained by use of BODIPY 493/503. The data are shown as a median fluorescence intensity (MFI) of *n* = 10–12 mice ± SEM. Gray bars = WT mice; turquoise bars = *Dgat1*KO mice. * *p* < 0.05, by two-way ANOVA, Tuckey post hoc test. **(B)** Accumulation of lipid droplets in WT and *Dgat1*KO neutrophils collected from bone marrow and incubated in vitro with 200 µM OA (left panel) and representative histograms of BODIPY 493/503 fluorescence (right panel). LDs were quantified by flow cytometry. The data are shown as a mean fluorescence intensity of *n* = 8 ± SEM; statistics were analyzed using *t*-test. **(C)** WT and *Dgat1*KO neutrophils collected from bone marrow and incubated in vitro with 200 µM OA, were harvested and analyzed using histology. Fluorescence microscopy images of neutrophils stained for LDs (green) and DNA (blue). Results are representative of at least three independent experiments

The main role of the DGAT1 enzyme is to catalyze the synthesis of TGs [14], which are accumulated in lipid droplets (LDs). Since diminished lipid (TGs)-dependent ATP production in DGAT1-deficient neutrophils could provide a plausible explanation for the decreased chemotactic capacity of neutrophils, we next determined whether a lack of DGAT1 impacts the formation of LD in neutrophils. We used the neutral lipid-specific dye BODIPY 493/503 to quantify lipids stored in LDs in neutrophils that infiltrates skin over the course of IMQ treatment. In agreement with a previous report [9], we noticed an increase in fluorescence, suggesting elevated levels of lipid storage in neutrophils as a result of skin inflammation (Fig. 4A). However, this difference was not robust. Moreover, we did not observe any significant differences in the biogenesis of LDs between neutrophils infiltrating the skin of WT and *Dgat1*KO mice. Taking into consideration that our experimental model may not provide enough stimulation for LD accumulation by neutrophils, we induced the formation of LD in neutrophils ex vivo by culturing the cells with oleic acid. Similarly to in vivo experiments, we observed no difference between WT and *Dgat1*KO neutrophils (Fig. 4B, C).

**Fig. 5:**
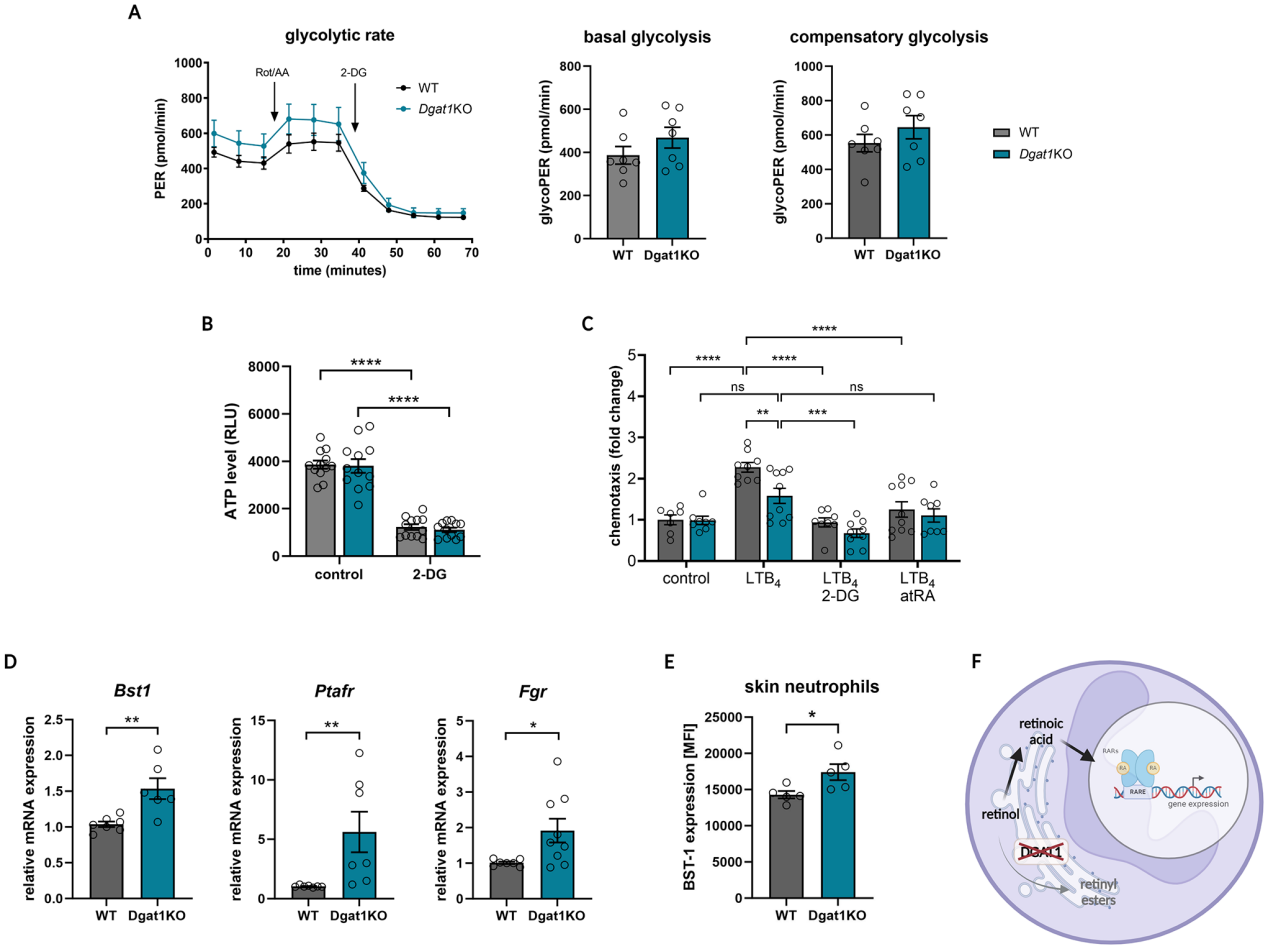
DGAT1 does not affect glycolytic ATP production, but regulates retinoic acid homeostasis. **(A)** Glycolytic rate, basal glycolysis and compensatory glycolysis of peripheral blood neutrophils of WT and *Dgat1*KO mice. The measurements of proton efflux rate (PER) were followed by sequential injections of rotenone (0.5 µM)/ antimycin A (0.5 µM) and 2-deoxy-D-glucose (2-DG) (50 mM). The data are shown as mean PER measurements at indicated time points of *n* = 7 ± SEM. Gray line = WT mice; turquoise line = *Dgat1*KO mice. **(B)** Total ATP level measured by luminescent assay for peripheral blood WT and *Dgat1*KO neutrophils with 50 mM 2-DG (glycolysis inhibitor). The data are shown as a mean of *n* = 9–12 ± SEM. **** *p* < 0.0001 by two-way ANOVA, Tuckey post hoc test. **(C) ** In vitro transwell chemotaxis assay for peripheral blood WT and *Dgat1*KO neutrophils with 100 nM leukotriene B_4_ (LTB_4_), as a chemoattractant, 50 mM 2-DG or 100 nM all-*trans* retinoic acid (atRA). The data are shown as a fold change related to control WT neutrophils. The mean of *n* = 7–10 ± SEM is shown. ** *p* < 0.01, *** *p* < 0.001, **** *p* < 0.0001 by two-way ANOVA, Tuckey post hoc test. **(D)** Relative expression of RA-responsive genes in activated neutrophils from sterile peritonitis (*n* = 6–9). The data are shown as a mean ± SEM; statistics were analyzed using *t*-test. (**E**) Relative expression of BST-1 protein presented as a median fluorescence intensity (MFI) on the surface of neutrophils infiltrating skin of WT and *Dgat1*KO mice after 3 days of IMQ treatment (*n* = 5). The data are shown as a mean ± SEM; * *p* < 0.05 by t-test. (**F**) Schematic presentation of DGAT1 role in the regulation of the gene expression of neutrophils

Since neutrophils mainly rely on glycolysis [40], we next measured the glycolytic rate and ATP production in WT and *Dgat1*KO mice. As expected, inhibition of electron transport chain components by rotenone and antimycin A resulted in only a slight increase in the proton efflux rate (PER) (Fig. 5A), because in mouse neutrophils as much as 90–95% of the total extracellular acidification comes from the glycolytic pathway. The addition of 2-deoxy-D-glucose (2-DG) (a glucose analog and glycolysis inhibitor) caused significant PER and ATP level reduction (Fig. 5A, B), confirming that glycolysis is the main source of ATP production in neutrophils [6]. However, we found that DGAT1 does not change the rates of basal or compensatory glycolysis (Fig. 5A), which was consistent with similar ATP production in WT and *Dgat1*KO neutrophils (Fig. 5B).

As expected, the addition of a 2-DG to the chemotaxis assay caused a reduction in neutrophil mobility to the baseline level. However, both WT and DGAT1-deficient neutrophils were similarly less responsive to LTB_4_ in the presence of 2-DG in the chemotaxis assay (Fig. 5C). These data suggest that DGAT1 in neutrophils is not responsible for the accumulation of TGs in LDs nor significantly affects glycolysis in neutrophils, and the inhibited chemotactic activity of *Dgat1*KO neutrophils is caused by different mechanism(s).

The less known activity of DGAT1 is the conversion of retinoic acid (RA) into retinyl esters, which prevents the accumulation of a non-physiological concentration of RA. Hence, the lack of DGAT1 results in a higher RA concentration [18]. Thus, RA accumulation might potentially affect neutrophil migration. To test this hypothesis, we cultured neutrophils with all-*trans* retinoic acid (atRA) and then performed a transwell chemotaxis assay with LTB_4_. Interestingly, the chemotactic activity of WT neutrophils cultured with atRA was reduced to a level comparable to that of neutrophils lacking DGAT1 (Fig. 5C). It is well-known that RA regulates transcription by interacting with nuclear RA receptors (RARs) and bound to RA response elements (RAREs) – DNA regulatory sequences located in the promoters or enhancers of target genes. Using RNAseq data (https://immgen.org) and a list of genes regulated by retinoids [31], we created a list of genes expressed in neutrophils and potentially regulated by RA (Suppl. Figure 4). Then, on the basis of the Gene Ontology (GO) of these genes, we selected those associated with chemotaxis and migration (*Bst1* - bone marrow stromal cell antigen 1, *Ptafr* - platelet-activating factor receptor, *Fgr* - feline gardner-rasheed sarcoma viral oncogene homolog), and analyzed their expression in the neutrophils of WT and *Dgat1*KO mice using a real-time qPCR method with specific primers. We observed a significantly elevated expression of these genes in activated neutrophils from *Dgat1*KO mice (Fig. 5D). We confirmed this at the protein level, demonstrating increased expression of BST-1 protein on *Dgat1*KO neutrophils infiltrating psoriatic skin (Fig. 5E). Taken together, these results suggest that DGAT1 expression in neutrophils mediates the conversion of retinoic acid into retinyl esters (Fig. 5F), and a lack of this enzyme results in the upregulation of RA levels in neutrophils, which in turn inhibits the chemotactic activity of neutrophils.

## Discussion

In recent years, there has been a growing interest in the regulatory role of cellular metabolism in the activity of immune cells. In this study we focus on the influence of DGAT1, an enzyme involved in TG synthesis, on neutrophil metabolism and chemotaxis. Despite the fact that DGAT1 was first described in 1998 [12, 13], its role in immune cells is still not well established. Macrophages from mice that overexpress *Dgat1* in adipose tissue demonstrate an increased lipid storage capacity [41]. On the other hand, DGAT1 inhibition causes a great reduction in the accumulation of external lipids in LDs of activated ILC2s [42]. DGAT1 has also been suggested to play a pivotal role in the differentiation of T cells by the control of lipid metabolism [43]. However, in our experiments we did not observe any difference in the accumulation of LDs in neutrophils from WT and *Dgat1*KO mice. Similarly, DGAT1 inhibitor (A922500) had no effect on the formation of LDs in unstimulated human neutrophils [44]. However, it is worth noting that neutrophils are not typical LD-accumulating cells. Human blood neutrophils may contain only 1 to 5 LDs per cell [9], which are usually undetectable in the physiological state, but increase in number during infection or inflammation [45].

Despite the fact that stored TGs can be broken down into fatty acids and glycerol, which can next be used in glycolysis, our data indicate that there is no difference in glycolysis and ATP production between WT and *Dgat1*KO neutrophils. Compared to other immune cells, where metabolic reprogramming generally ensures differentiation into distinct subtypes, neutrophils modulate their metabolic pathways to execute different effector functions, such as chemotaxis, generation of ROS, formation of NETs and degranulation [46]. Neutrophils are primarily glycolytic cells, but other metabolic pathways are also important for their pro-inflammatory activity [46, 47]. For a long time, it was believed that migrating neutrophils preferred to uptake glucose to produce ATP during glycolysis as a main source of energy used in chemotaxis [48]. However, as has been shown in recent studies, chemotaxis is only partially dependent on the energy generated during glycolysis [46, 47]. On the basis of our results and data shown by Sadiku et al. [49], that neutrophils generate energy stores in the form of glycogen, which are dynamically regulated by gluconeogenesis and glycogenesis during their life cycle [49], it is likely that neutrophils primary store energy in the form of glycogen and not in LDs, and DGAT1 does not change neutrophil activity such as chemotaxis though lipid metabolism.

Our results also suggest that the acyl-CoA:retinol acyltransferase (ARAT) activity of DGAT1 might be more important for neutrophils than its basic acyl-CoA:diacylglycerol acyltransferase activity. The main role of DGAT1 in retinoid homeostasis is not unprecedented in the context of the regulation of the function of immune cells. For example, the DGAT1-mediated synthesis of retinol esters and the prevention of RA formation has been shown to regulate T cell differentiation in a mouse model of multiple sclerosis [50]. DGAT1 participated in the polarization of T cells into retinol-dependent Treg cell formation, which attenuated experimental autoimmune encephalomyelitis. Therapeutic administration of RA has also been shown to regulate cytokine expression [51] and have anti-inflammatory effects in dermatological diseases, such as psoriasis [21], at least partially by inhibiting the pro-inflammatory activity of neutrophils [22]. The anti-inflammatory effects of retinoids are well established, including the inhibitory effect of retinoids on neutrophil migration [23]. However, the molecular mechanism underlying the action of retinoids on neutrophils is still not clear. Retinoids act on target cells and bind to retinoid nuclear receptors, which are transcription factors that control the expression of specific genes which possess RA response elements (RARE) within the promoters [52]. Physiologically, the concentration of retinoic acid within tissues is generally very low [53], and even a slight increase in its concentration can modulate cell functions. DGAT1 has ARAT activity in vitro [17, 54] and acts physiologically as an ARAT in the murine intestine [55, 56] and the skin [18], and as such, it plays a significant role in maintaining retinoid homeostasis. A lack of DGAT1 is correlated with an increased RA concentration, which in turn can modulate the immune response during inflammation.

We have shown inhibited neutrophil recruitment to IMQ-induced psoriatic skin in *Dgat1*KO mice, and reduced the chemotaxis of neutrophils lacking DGAT1 in the presence of LTB_4_. LTB_4_ is a lipid chemoattractant that plays an important role in neutrophil recruitment to psoriatic skin [57]. Physiologically, in human neutrophils LTB_4_ is inactivated mainly by hydroxylation catalyzed by the cytochrome P450 enzyme CYP4F3A [58, 59], and this hydroxylation activity is strongly induced by atRA [60]. Furthermore, RA inhibits LTB_4_ production by neutrophils [61] and increases the gene and protein expression of CYP4F enzymes in human keratinocytes from psoriatic patients [62]. Topical administration of retinoids (mainly RA) reduces LTB_4_-induced neutrophil migration into human skin [63]. Interestingly, the reduced infiltration of neutrophils after DGAT1 inhibition has also been observed in granuloma tissue [64], but the authors of this research attribute this more to reduced neutral lipid accumulation in necrotic granulomas, which leads to improved control of bacterial burden and, as a result, lower neutrophil recruitment. Thus, our data are the first that demonstrate the direct effect of DGAT1 on neutrophil functions.

In conclusion, although DGAT1 is normally associated with lipid metabolism, here we have shown that the ARAT activity of DGAT1 plays a more significant role in the regulation of murine neutrophil chemotactic activity. The precise molecular mechanism of this action and influence on other pro-inflammatory functions require future in-depth research, but our data already suggest the therapeutic potential of DGAT1 inhibitors in the treatment of diseases associated with excessive neutrophil infiltration.

## Electronic supplementary material

Below is the link to the electronic supplementary material.

Supplementary Material 1

## Data Availability

All data generated or analyzed during this study are included in this published article and its supplementary information. The original experimental raw data can be obtained from the corresponding author upon reasonable request.
