# Supplementary material for: Lazy neutrophils – a lack of DGAT1 reduces the chemotactic activity of mouse neutrophils
Source: Inflamm Res. 2024 Jul 24;73(10):1631–43. doi: 10.1007/s00011-024-01920-6 (PMC11445369; doi:10.1007/s00011-024-01920-6)
Supplement: Supplementary file 1 — Supplementary Material 1 [file 11_2024_1920_MOESM1_ESM.docx]

**Supplementary material**


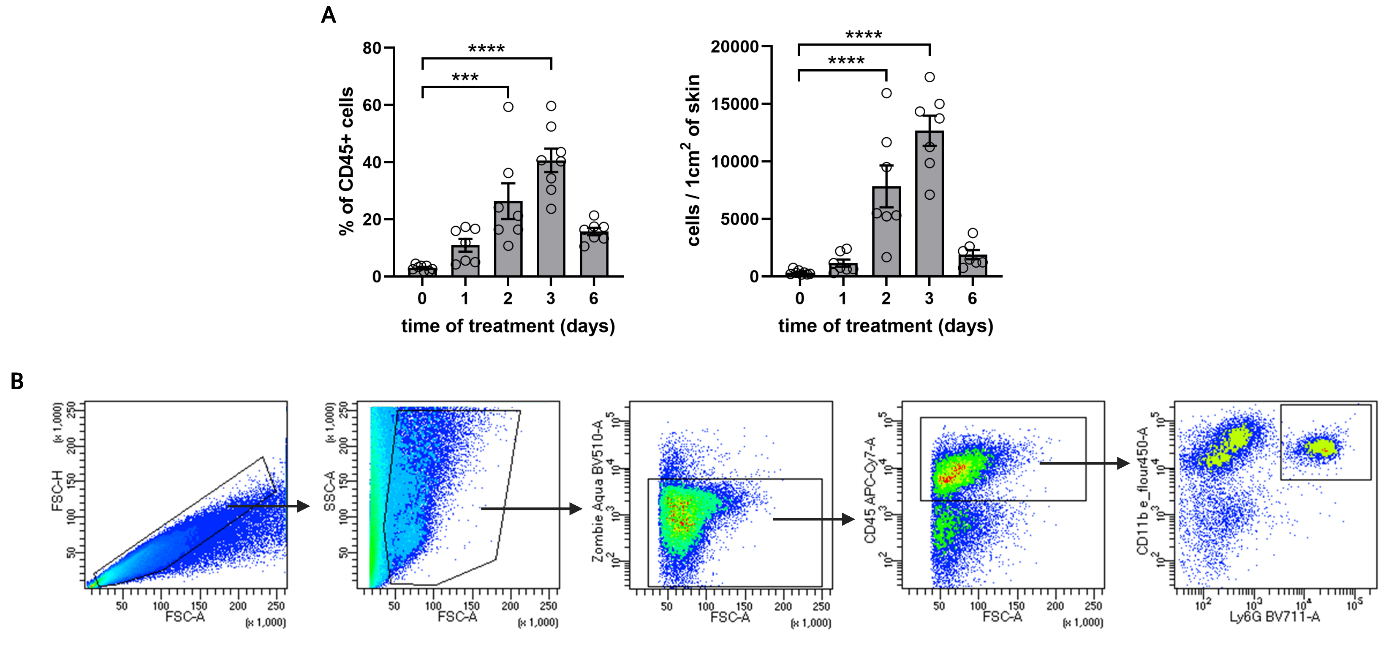


**Suppl. 1 Kinetics of neutrophil infiltration to the psoriatic skin of IMQ-treated mice.** WT mice were treated with IMQ to induce psoriasis-like dermatitis. Skin biopsies were harvested and subjected to flow cytometry analysis. Total leukocytes were detected using anti-CD45 mAbs, whereas neutrophils were detected using anti-Ly6G and -CD11b mAbs. **(A)** Data are shown as a percentage of neutrophils among CD45 + cells (left panel) and as an absolute number of neutrophils in 1 cm^2^ of skin (right panel). The data are shown as a mean of n = 7 mice ± SEM. *** p < 0.001, **** p < 0.0001 by two-way ANOVA, Tuckey post hoc test. **(B)** Gating strategy for neutrophil evaluation used in all flow cytometry analyses. FSC-H/FSC-A gating excluded doublets, FSC/SSC gating excluded debris, Zombie Aqua was used to exclude dead cells, CD45 was used to gate leukocytes, and CD11b and Ly6G to define neutrophils.


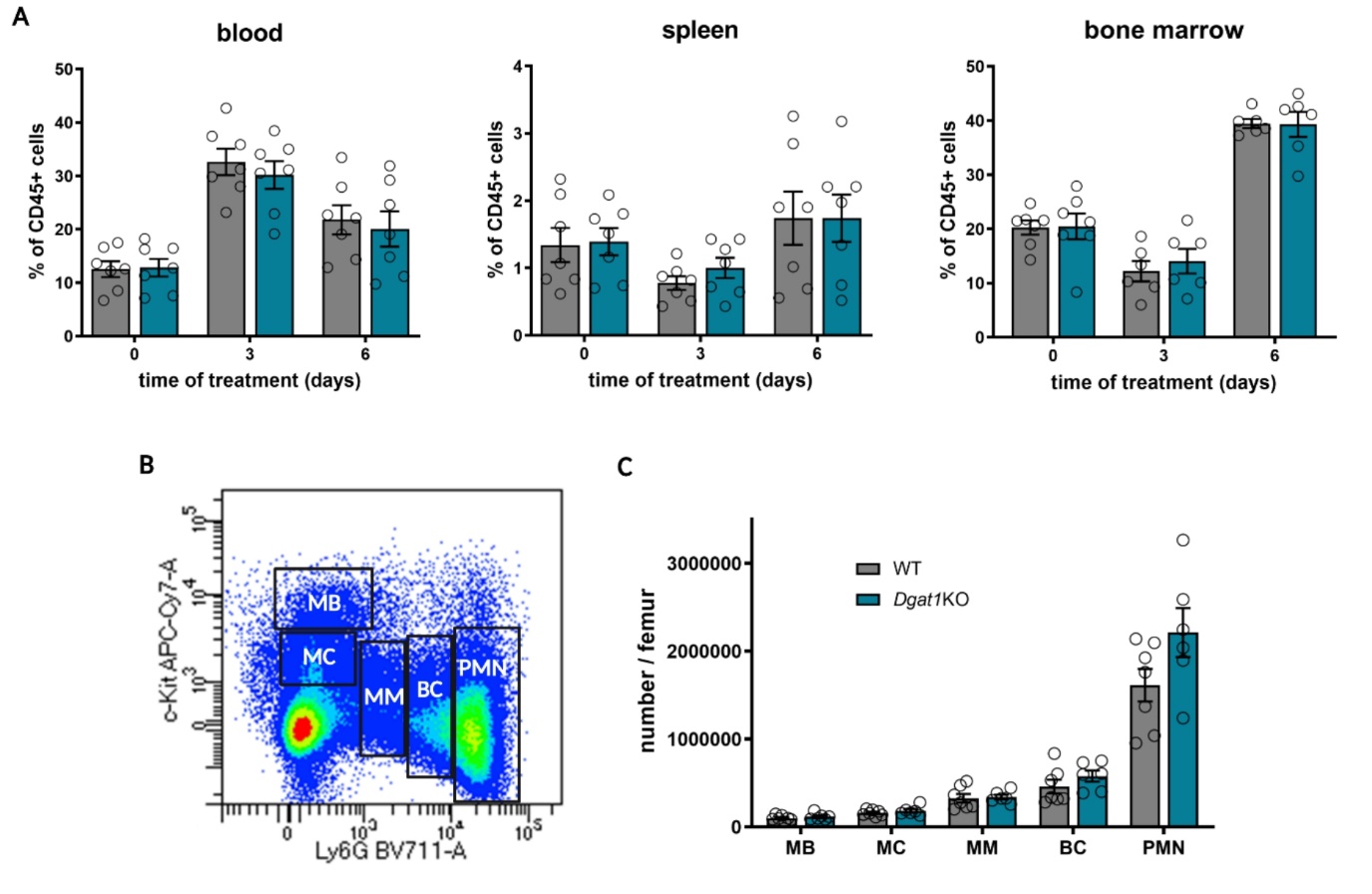


**Suppl. 2 Lack of systemic differences between WT and *Dgat1*KO neutrophils. (A)** Kinetic changes in neutrophil frequency in blood, spleen and bone marrow from IMQ-treated WT and *Dgat1*KO mice. Total leukocytes were detected using anti-CD45 mAbs, whereas neutrophils were detected using anti-Ly6G and anti-CD11b mAbs. Data are shown as a percentage of neutrophils among CD45 + cells. The data are shown as a mean of n = 6–8 mice ± SEM; statistics were analyzed using by two-way ANOVA, Tuckey post hoc test. Gray bars = WT mice; turquoise bars = *Dgat1*KO mice. **(B)** Gating strategy used to enrich populations of MBs (myeloblasts), MCs (myelocytes), MMs (metamyelocytes), BCs (band cells), and PMNs (polymorphonuclear neutrophils). **(C)** Flow cytometric analysis of untreated WT and *Dgat1*KO mice granulopoiesis. Data are shown as absolute number of neutrophils in one femur. The data are shown as a mean of n = 7 mice ± SEM; statistics were analyzed by *t*-test. Gray bars = WT mice; turquoise bars = *Dgat1*KO mice.


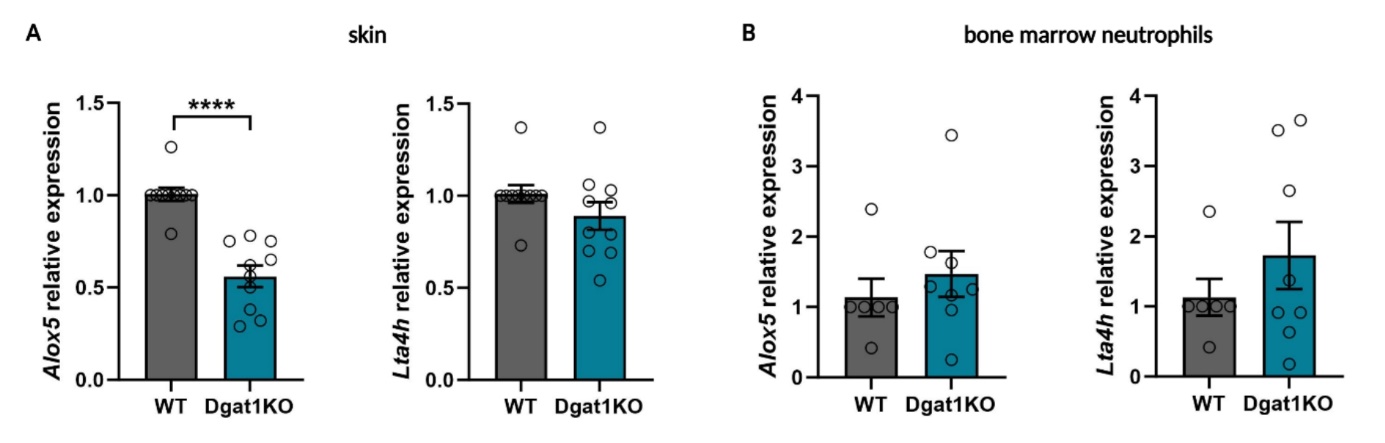


**Suppl. 3 Expression of genes involved in LTB_4_ synthesis. (A)** *Alox5* and *Lta4h* genes expression in the skin of WT and *Dgat1*KO mice. The data are shown as a mean of n = 10 mice ± SEM. Gray bars = WT mice; turquoise bars = *Dgat1*KO mice. **** p < 0.0001 by *t*-test. **(B)** *Alox5* and *Lta4h* genes expression in bone marrow neutrophils (n = 6–8). The data are shown as a mean ± SEM; statistics were analyzed using *t*-test.


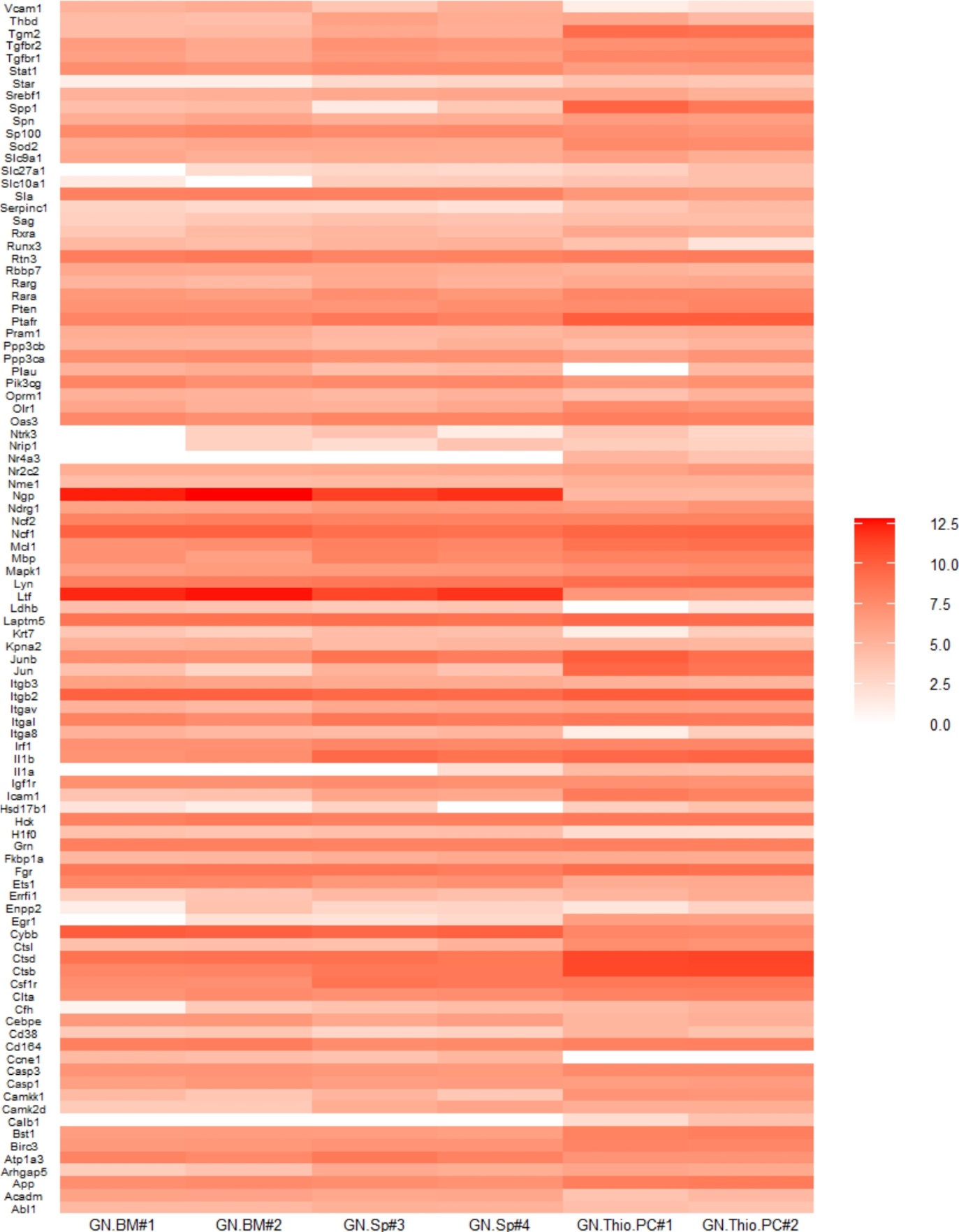


**Suppl. 4 Expression of RA-regulated genes in neutrophils.** RNA-Seq based analysis of expression of genes regulated by retinoids in murine neutrophils from bone marrow (GN.BM), spleen (GN.Sp) and thioglycollate-induced peritonitis (GN.Thio.PC). Heatmap was created based on the list of genes regulated by retinoids (31), and shows log2-transformed normalized gene counts from Immunological Genome Project database.
